# Spheroid body-forming cells in the human gastric cancer cell line MKN-45 possess cancer stem cell properties

**DOI:** 10.3892/ijo.2012.1720

**Published:** 2012-11-29

**Authors:** JIANMING LIU, LILIN MA, JUNFEI XU, CHUN LIU, JIANGUO ZHANG, JIE LIU, RUIXIN CHEN, YOULANG ZHOU

**Affiliations:** 1 Department of General Surgery, Affiliated Hospital, Nantong University, Nantong 226001, Jiangsu Province, P.R. China; 2 Laboratory Animal Center, Nantong University, Nantong 226001, Jiangsu Province, P.R. China; 3 Department of Pathology, Nantong University, Nantong 226001, Jiangsu Province, P.R. China; 4 Research Center of Clinical Medicine, Affiliated Hospital, Nantong University, Nantong 226001, Jiangsu Province, P.R. China

**Keywords:** gastric cancer, cancer stem cell, CD44, Oct4, Sox2, Nanog

## Abstract

The cancer stem cell theory hypothesizes that cancer stem cells (CSCs), which possess self-renewal and other stem cell properties, are regarded as the cause of tumor formation, recurrence and metastasis. The isolation and identification of CSCs could help to develop novel therapeutic strategies specifically targeting CSCs. In this study, we enriched gastric cancer stem cells through spheroid body formation by cultivating the human gastric cancer cell line MKN-45 in defined serum-free medium. The stemness characteristics of spheroid body-forming cells, including self-renewal, proliferation, chemoresistance, tumorigenicity of the MKN-45 spheroid body-forming cells were evaluated, and the expression levels of stemness genes and related proteins in the MKN-45 spheroid body-forming cells were assessed. Furthermore, immunofluorescence staining for the stem cell markers on spheroid body-forming cells was examined to evaluate the association between stemness factors (Oct4, Sox2, Nanog) and the proposed CSC marker CD44. Our data demonstrated that non-adherent spheroid body-forming cells from the gastric cancer cell line MKN-45 cultured in stem cell-conditioned medium possessed gastric CSC properties, such as persistent self-renewal, extensive proliferation, drug resistance, high tumorigenic capacity and overexpression of CSC-related genes and proteins (Oct4, Sox2, Nanog and CD44), compared with the parental cells. More importantly, CD44-positive cells co-expressing the pluripotency genes Oct4, Sox2 and Nanog may represent gastric CSCs. Further experiments using more refined selection criteria such as a combination of two or multiple markers would be useful to specifically identify and purify CSCs.

## Introduction

Gastric cancer is one of the most common cancers worldwide, and mortality due to gastric cancer is second next to lung cancer (1). Although surgery is the standard treatment of localized gastric cancer, the results are often disappointing, with recurrence rates as high as 70% after successful complete (R0) resection. Attempts to improve outcome with adjuvant therapy have yielded only modest success (2).

Emerging evidence indicates that cancer stem cells (CSCs) may be involved in tumor maintenance, therapy resistance, tumor progression, and distant metastasis (3,4). CSCs are defined as a subpopulation of cells within a tumor that possess the capacity for self-renewal and that can cause the heterogeneous lineage of cancer cells that constitute the tumor (5). The most important issue in the research of CSCs is how to isolate and identify CSCs. Some research groups have reported that gastric CSC fractions could be successfully enriched by some cell surface phenotypes, specifically CD44, epithelial cell adhesion molecule (EpCAM) (6,7). Nevertheless, these markers, are not specific for identifying gastric CSCs (6). So far, no markers for putative gastric CSCs have yet been generally accepted, and further study is needed to explore the isolation method for gastric CSCs.

As a functional approach, spheroid body formation is particularly useful to enrich the potential CSC subpopulations when the specific CSC makers have not been defined, as is the case for most CSCs (8–10). Therefore, in the present study we developed spheroid body-forming cells in gastric cancer cell line MKN-45 and determined whether these cells acquired CSCs characteristics, including self-renewing capacity, chemoresistance and tumorigenic capacity.

## Materials and methods

### Culture of parental, spheroid body-forming cells

The human gastric cancer cell line MKN-45 obtained from the Cell Bank of the Chinese Academy of Sciences, Shanghai, China was cultured in RPMI-1640 medium containing 10% fetal bovine serum (FBS) and plated at the density of 1×10^6^ live cells per 75-cm^2^ flask. When the cells attached, we passaged them upon confluence. Spheroid bodies were derived by placing the parental cells into serum-free RPMI-1640 culture medium containing 1% N-2 supplement, 2% B-27 supplement (Invitrogen), 1% antibiotic mixture (Gibco), 20 ng/ml human FGF-2 and 100 ng/ml EGF (Chemicon). The parental cells were plated in 96-well ultra-low attachment plate (Corning) at 100 cells per well. Two weeks later, plates were analyzed for spheroid body formation and were quantified using an inverted microscope (Olympus) at ×40 and ×100 magnification. After primary spheroid body reached the size of approximately 200–500 cells per spheroid body, the spheroid bodies were dissociated at the density of 1,000 cells per milliliter and 100 single cell suspension (100 *μ*l) was seeded in each well of a 96-well ultra-low attachment plate (Corning) in serum-free medium described above. Two weeks later, wells were analyzed for subspheroid body formation.

### Quantitative real-time PCR

Total-RNA was extracted from the parental cells, spheroid body-forming cells using Qiagen RNeasy mini kit (Qiagen) according to the manufacturer’s instructions. RNA was treated with DNase I (Qiagen) to eliminate genomic DNA contamination. The integrity and purification of RNA samples were monitored by agarose gel electrophoresis. The concentration of RNA was determined by repeated OD measurements of aliquots at a wavelength of 260 nm. Reverse-transciption reaction to transcript 1 *μ*g total-RNA into complementary DNA was performed with reagents of synthesis system (Qiagen).

To determine fold changes in each gene, real-time qPCR was performed using the Eppendorf Mastercycler ep realplex (2S; Eppendorf, Hamburg, Germany). EvaGreen (Biotium Inc., Hayward, CA) served as a dye that binds to amplified DNA to emit fluorescence during reactions. EvaGreen is an optimal green fluorescent DNA dye for qPCR. The reaction mixture of 25 *μ*l contained 12.5 *μ*l of EvaGreen™ qPCR Master mix (Biotium Inc.), 1 *μ*l of primers (10 mM) and 1 *μ*l of template cDNA, and 10.5 *μ*l of double distilled water (ddwater). The EvaGreen (Biotium Inc.) served as a dye that binds to amplified DNA to emit fluorescence during reactions. The glyceraldehyde-3′ phosphate dehydrogenase (GAPDH) gene served as an internal control for expression levels of target apotosis genes. The primer sequences and PCR conditions are summarized in Table I. After an initial incubation for 2 min at 96°C, the reactions were carried out for 40 cycles at 96°C for 15 sec and 60°C for 45 sec (fluorescence collection). Fluorescent readings were taken during the extension step of each cycle. Melting curve analysis was performed to ensure the amplification of a single PCR production. Reactions with no template was included as negative control. By setting the threshold at the level at the middle steady portion of reaction cycles versus florescence curve, the Ct values of target genes were calculated using customized software, and the 2(−ΔΔC(T)) method was used. Finally, the PCR products were separated on 1.5% agarose gel electrophoresis in the presence of ethidium bromide, and visualized on an ultra-violet illuminator to verify product sizes, and recorded. The qPCRs were performed three times in triplicate.

### Immunofluorescence staining for stem cell markers

In brief, cells plated onto poly-L-lysine-coated glass coverslips were fixed with 4% paraformaldehyde and then washed with PBS. Cells were permeabilized with 0.1% Triton X-100/PBS for 10 min. Consequently, the cells were incubated with primary antibodies (Oct-4, Nanog, Sox2 or CD44; Santa Cruz Biotechnology). Cells were further probed with fluorescein isothiocyanate or Rhodamine-tagged secondary antibodies. The fluorescence was recorded by inverted fluorescence microscope (Leika).

### Western blot analysis

For western blot analyses, protein was harvested from cells plated to 70 to 80% confluence. Spheroid body-forming cells or parental cells were lysed directly in lysis buffer to collect whole cell extracts. Protein samples for western blot analysis were prepared by boiling after the addition of denaturing sample buffer. Then, proteins were separated using SDS-PAGE on an 8 or 15% gel, transferred onto PVDF membranes. Membranes were incubated at 4°C overnight with primary antibody, and subsequently incubated with horseradish peroxidase-conjugated secondary antibodies for 1 h at room temperature. Finally, protein bands were visualized using chemiluminescence (Santa Cruz Biotechnology) exposure on BioMax film (Kodak). Concentrations used for primary antibodies were: anti-CD44 1:200, anti-Oct4 1:200, anti-Sox2 1:200, anti-Nanog 1:200 (all from Santa Cruz Biotechnology).

### Chemoresistance assay

Rates of resistance to drugs were assessed using MTT assay. Briefly, 2,000 healthy spheroid body-forming cells or parental cells per well were plated in 96-well plates in 100 *μ*l RPMI-1640 medium (4 wells per group) with chemotherapeutic drugs (5-Fu, DDP) or control PBS. At each time point (24 and 48 h), 10 ml MTT solution was added to each well and the plate was incubated for 4 h at 37°C, then the medium was replaced by 150 ml DMSO. To assess the effect of drug resistance of spheroid body-forming cells or parental cells, we treated the dissociated cells with 5-Fu (50 *μ*g/ml, 100 *μ*g/ml, 200 *μ*g/ml) alone, DDP (10 *μ*g/ml, 20 *μ*g/ml, 40 *μ*g/ml) alone, 5-Fu plus DDP (50 *μ*g/ml + 10 *μ*g/ml; 100 *μ*g/m + 20 *μ*g/ml; 200 *μ*g/ml + 40 *μ*g/m) or control PBS for 24 and 48 h. MTT assay is based on mitochondrial conversion of MTT to yellowish formazan, being indicative of the number of viable cells. The number of viable cells was evaluated by absorbance OD450 nm (Abs) using Model 680 microplate reader.

### In vivo tumorigenicity experiments

Male athymic nude mice (nu/nu), 6 to 8 weeks old, were obtained from Shanghai Laboratory Animal Center, Chinese Academy of Sciences, Shanghai, China, and were housed under pathogen-free conditions in the barrier animal facility. All animal procedures were carried out with the approval of the Animal Ethics Committee of Nantong University.

For *in vivo* tumorigenicity experiments, equal number (1×10^4^, 2×10^4^, 2×10^5^, 2×10^6^) of freshly dissociated cells was suspended in 200 *μ*l PBS, the spheroid body-forming cells were injected subcutaneously into the right rear flank of each mouse (6 mice per group) and the parental cells were injected subcutaneously into the left rear flank of each mouse, we examined the tumorigenic capacity of spheroid body-forming cells and parental cells. The mice were observed for tumor growth every 10 days over 8 weeks and then sacrificed by cervical dislocation. The grafts were removed, fixed with 10% buffered formalin, and stained with hematoxylin and eosin (H&E).

### Statistical analysis

All experiments were repeated at least three times and representative results are presented. All values in the figures and text are the means ± SD. Statistical analyses were performed using the SPSS statistical software package (SPSS/PC+, SPSS Inc., Chicago, IL, USA). Any significant differences among mean values were evaluated by the Student’s t-test. A two-sided p<0.05 was accepted as significant.

## Results

### Gastric cancer cells form anchorage-independent spheroid bodies

**Figure 1.**
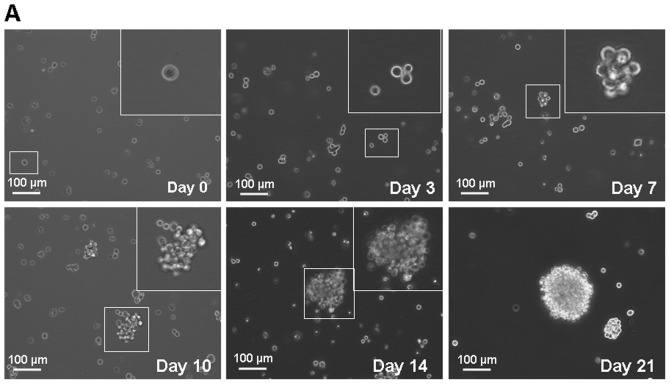

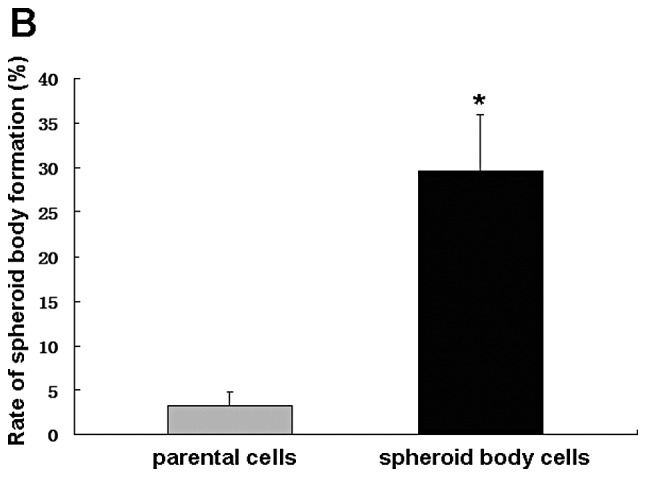
MKN-45 cells formed the anchorage-independent, self-renewing spheroid bodies. (A) Generation of a spheroid body from a single MKN-45 cell. The propagation of a single cell cultured in a 96-well dish was recorded at day 0, 3, 7, 10, 14 and 21, separately (×200 magnification). (B) The sub-spheroid body formation rate of spheroid body cells were also higher than that of parental cells (^*^p<0.01).

MKN-45 parental cells were cultured in serum-free medium described in the methods section. In this condition, cells grew as non-adherent, three-dimensional spheroid clusters, called spheroid body. The self-renewing capacity of these spheroid body-forming cells was assessed by dissociating them into single cell and growing in serum-free medium described in the methods section. Fig. 1A shows the process of single MKN-45 cell forming a spheroid body. After 2 weeks, these cells derived from spheroid body-forming cells generated sub-spheroid bodies again at 29.70±6.21% compared with 3.30±1.49% of parental cells (Fig. 1B).

### Spheroid body-forming cells possess the ability of resistance to conventional chemotherapy in vitro

**Figure 2.**
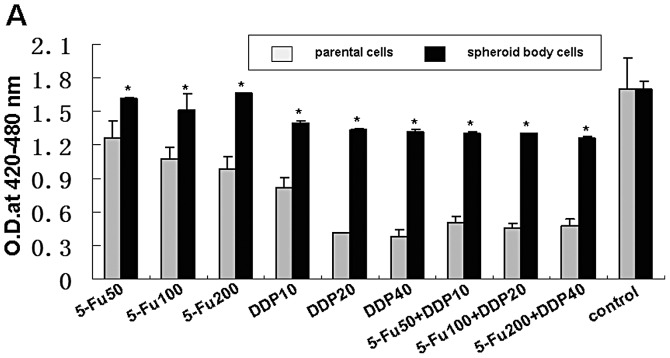

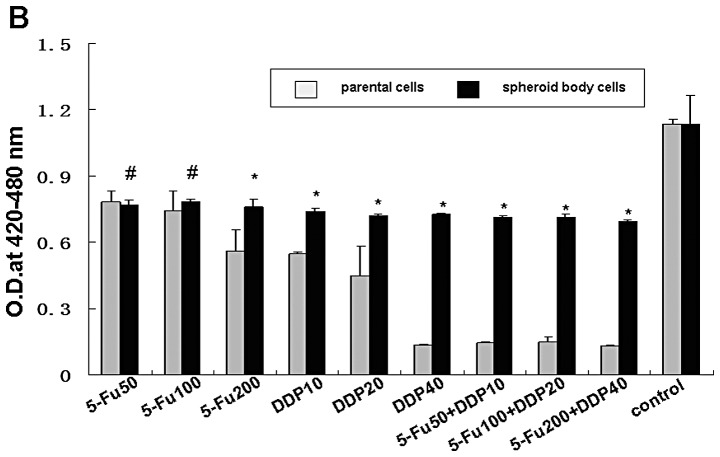
Spheroid-forming cells proliferate extensively and possess resistance ability to conventional chemotherapeutics *in vitro*. The MKN-45 spheroid body-forming cells showed a drug resistance phenotype. The parental cells and spheroid body-forming cells were treated with different concentrations (*μ*g/ml) of 5-Fu and DDP at the beginning of plating for (A) 24 or (B) 48 h. Cell survival was determined by MTT assay (^#^p>0.05; ^*^p<0.01).

The MKN-45 spheroid body-forming cells exhibited general resistance to 5-Fu and DDP in the treatment of 24 h (Fig. 2A). Compared with the parental MKN-45 cells, the survival rates of spheroid body-forming cells were higher under the treatment of 50 *μ*g/ml, 100 *μ*g/ml and 200 *μ*g/ml 5-Fu (1.3-fold, 1.4-fold and 1.7-fold, respectively); 10 *μ*g/ml, 20 *μ*g/ml and 40 *μ*g/ml DDP (1.7-fold, 3.2-fold and 3.4-fold, respectively). Whereas, under the treatment for 48 h (Fig. 2B), the relative survival rates were not significantly increased, neither 5-Fu nor DDP. But in the treatment of 5-Fu combined with DDP, the survival rates of MKN-45 spheroid body-forming cells were increased 2.6-fold, 2.9-fold and 2.7-fold, respectively, under the treatment for 24 h; and 5.1-fold, 4.8-fold and 5.4-fold, respectively, for 48 h.

### Spheroid body-forming cells overexpress gastric CSC-related genes and proteins

**Figure 3.**
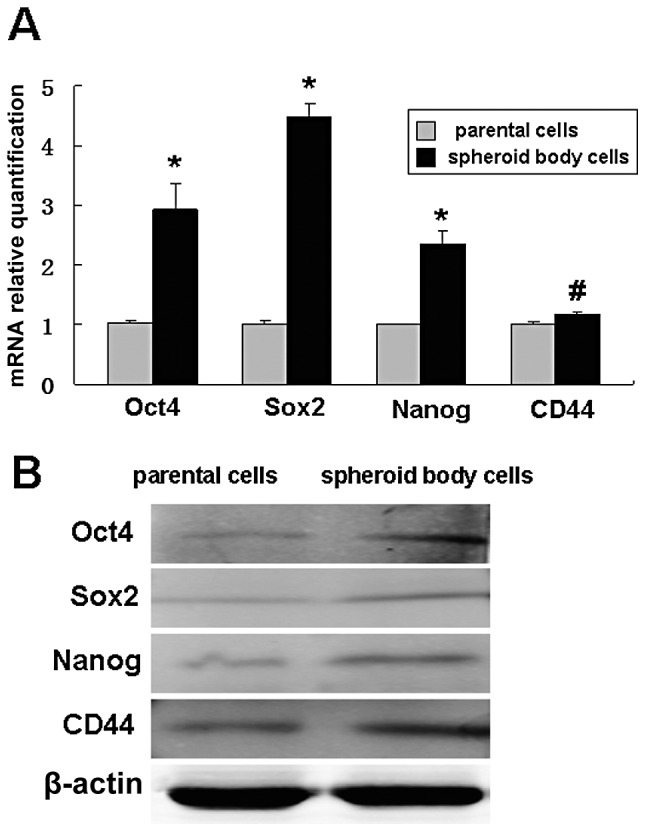
Spheroid body-forming cells overexpress gastric CSC related proteins and genes. (A) Quantitative real-time PCR analysis showed the elevated expression of Oct-4, Sox2, Nanog and CD44 genes in the MKN-45 spheroid body-forming cells compared with the parental cells (^*^p<0.01; ^#^p<0.05). (B) Western blot analysis showed the gastric CSC related proteins (Oct4, Sox2, Nanog and CD44), and the expression of these CSC-related proteins in spheroid body cells were higher than that of parental cells.

Quantitative real-time PCR and western blot analysis were performed on spheroid body-forming cells and parental cells. The results showed that the cells expressing Oct-4, Sox2, Nanog and CD44 were significantly more in spheroid body-forming cells than in parental cells (Fig. 3).

### Intracellular localization of Oct-4, Sox2, Nanog and CD44 in spheroid body-forming cells

**Figure 4.**
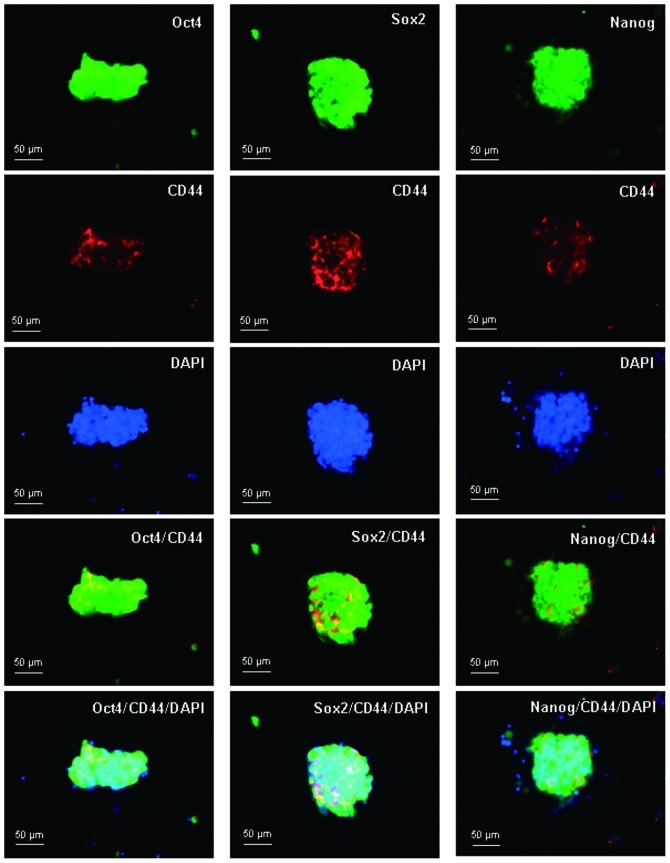
Intracellular localization of Oct-4, Sox2, Nanog and CD44 by immunofluorescence staining. Dual staining of Oct-4/CD44, Sox2/CD44 and Nanog/CD44 indicated that CD44 positively stained cells were co-stained with either Oct-4, Sox2 or Nanog.

To examine the subcellular localization of Oct-4, Sox2, Nanog and CD44 in spheroid body-forming cells, immunofluorescent staining of Oct-4, Sox2, Nanog and CD44 was performed. Oct-4, Sox2 and Nanog proteins were positively stained within the perinuclear and cytoplasm of the spheroid body-forming cells, and CD44 was positively stained mainly in the membrane. Dual staining of Oct-4/CD44, Sox2/CD44, and Nanog/CD44 indicated that the embryonal proteins (Oct-4, Sox2 and Nanog) were colocalized with CD44 in the spheroid body-forming cells (Fig. 4).

### Spheroid body-forming cells exhibit high tumorigenicity in vivo

**Figure 5.**
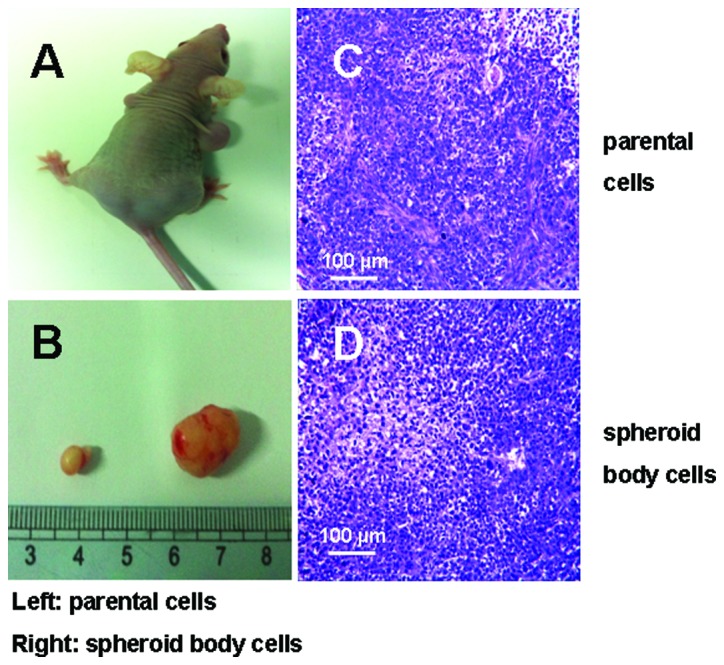
Spheroid body-forming cells exhibited high tumorigenicity *in vivo*. (A) The representative examples of xenograft tumors formed after subcutaneous injection with 2×10^6^ MKN-45 parental cells and spheroid-forming cells, separately. (B) The nodules formed by injecting 2×10^6^ parental cells and sphere-forming cells, separately. (C and D) H&E staining revealed that the histological features of xenograft tumors induced by the MKN-45 spheroid body-forming cells were similar to those induced by the parental cells.

The tumorigenicity experiments *in vivo* showed that as few as 2×10^4^ cells from the MKN-45 spheroid body were able to form a tumor when subcutaneously injected into nude mice, while 2×10^6^ parental cells were needed (Fig. 5A and B). This was 100 times higher than that of spheroid body-forming cells. Moreover, spheroid body-forming cells generated subcutaneous tumors with larger volume and shorter time compared with those generated from parental cells. H&E examination of xenografts derived from MKN-45 spheroid body-forming cells showed that these tumors closely resembled tumors from the parental cells, mainly containing differentiated cells (Fig. 5C and D).

## Discussion

The hypothesis that cancers are maintained by a subpopulation of stem cells while non-stem cells have a finite life span raises the possibility that targeting cancer stem cells could provide a means of cancer control. As a preliminary step to investigate whether this hypothesis is applicable to gastric cancers, it is necessary to identify and isolate gastric cancer stem cells, which could provide new insight into the gastric cancer tumorigenic process and possibly bear great therapeutic implications.

Three distinct methodologies based on the properties of CSC have been successfully used for isolation of CSC from solid tumors (5,11,12). i) Fluorescence-activated cell sorting (FACS) according to CSC-specific cell surface markers such as CD44 or CD133 is made possible for isolation of CSCs (4,13,14). ii) The side populations (SP) of tumor cells, which display intracellular Hoechst 33342 exclusion *in vitro*, also may cause chemoresistance is isolated and characterized as CSCs (15–17). iii) The spheroid body formation assay in which cells are cultured in non-adherent condition in a serum-free medium supplemented with basic fibroblast growth factor (bFGF) and epidermal growth factor (EGF) is a practical approach for individual solid tumor tissues or cancer cells (18,19).

There have been some reports on the isolation and identification of gastric CSCs by FACS based on CD44, which is a proposed marker for gastric CSCs (6,7), however, the sensitivity and specificity for identifying gastric CSCs are being challenged. For example, Rocco *et al*(20) reported although CD133(+) and CD133(+)/CD44(+) were detectable in human primary gastric cancers, they neither expressed stem-like properties nor exhibited tumor-initiating properties in xenograft transplantation experiments. The sorting of SP cells is another type of method for the isolation and identification of gastric CSCs (21). However, some studies have indicated that there was not a significant association between the SP fraction and CSCs. Patrawala *et al*(22) reported that glioma cell lines which expressed ABCG2, an ATP-binding cassette half-transporter that is associated with SP cells, had a similar tumorigenicity as ABCG2-negative cells. Burkert *et al*(23) also reported that among four colon cancer cell lines examined, SP and non-SP cells were similarly clonogenic *in vitro* and tumorigenic *in vivo* and displayed equivalent multipotential differentiation potential. They also showed that SP and non-SP populations are interconvertible, each giving rise to the other in culture. Takaishi *et al*(6) found in their study that human gastric cancer MKN-45 cells have a significant SP fraction, but the SP and non-SP cells both possess tumorigenic ability *in vitro* and *in vivo*.

Spheroid body culture has been increasingly used as a method for enriching stem cells which relies on their property of anchorage-independent growth. Various types of potential CSC subpopulations from primary tumors have been reported to be isolated and enriched by the application of spheroid body culture (24–26). The spheroid body-forming cells from primary tumors, such as ovarian cancer and breast cancer, showed stem-like properties and expressed their CSC markers (24,27). To our knowledge, there have been few reports on the isolation and characterization of gastric CSCs by the method of spheroid body culture, therefore, we developed spheroid body cells by cultivating human gastric cancer cell line MKN-45 in defined serum-free medium, and demonstrated that those cells derived from spheroid body could generate greater numbers of new spheroid bodies than the parental cells, indicating that spheroid body-forming cells were capable of self-renewal and proliferation, which is an important characteristic of CSCs.

Chemoresistance is another important characteristic of CSCs. To assess whether the self-renewing spheroid body-forming cells possess a hypothesized CSC chemoresistant property, we examined the sensitivity of spheroid body-forming cells to chemotherapeutics. The MKN-45 spheroid body-forming cells exhibited general resistance to 5-Fu and DDP, even in the treatment of 5-Fu combined with DDP, and showed higher survival percentages compared with its parental cells. These results support a role for these spheroid body-forming cells in gastric cancer chemoresistance, which may explain why current therapies fail to eradicate cancer cells and prevent tumor re-growth.

Xenotransplantation is generally regarded as the gold standard for evaluating tumorigenicity of tumor cells. We tested the MKN-45 spheroid body-forming cells for their tumor initiating capability. *In vivo*, as fewer as 100-fold cells from spheroid body-forming cells could generate tumors upon xenotransplantation than those from parental cells. Moreover, spheroid body-forming cells generated subcutaneous tumors with larger volume and shorter time compared with those generated from parental cells. These data therefore indicated that the spheroid body-forming cells represented CSCs that had tumorigenic capacity.

To further explore the CSC properties of spheroid body-forming cells, We evaluated the MKN-45 spheroid body-forming cells for their stemness characteristics. Overexpression of stem cell-specific transcription factors such as Oct4, Sox2 and Nanog is a vital characteristics of CSCs (28). These transcription factors often function in combinatorial complexes to regulate the expression of gene loci which are involved in self-renewal, proliferation and differentiation (29). CD44, which is a type I transmembrane glycoprotein that serves as the receptor for the extracellular matrix component, hyaluronic acid, was one of the first markers of solid tumors that was shown to be enriched in tumor-initiating cells (13). Recent studies have provided support for its role as a CSC marker (6,30). In this study we found that all three transcription factors (Oct4, Sox2 and Nanog) and CD44 are overexpressed in MKN-45 spheroid body-forming cells as compared with parental cells. More importantly, we have first focused on the question of whether there is a physical linkage between CD44 and the three transcription factors in spheroid body-forming cells. we found that the CD44 positive stained spheroid body-forming cells were costained with Oct4, Sox2 or Nanog, indicating CD44 positive cells co-expressed of the pluripotency factors Oct4, Sox2 or Nanog in MKN-45 spheroid body might represent a kind of gastric CSCs.

In summary, the study demonstrated that the non-adherent spheroid body-forming cells from human gastric cancer cell line MKN-45, which are cultured in stem cell conditioned medium, possess gastric CSC properties. Further experiments using more refined selection criteria such as a combination of two or multiplemarkers would be useful to specifically identify and purify CSCs. Testing on resected tumor samples or peritoneal effusion fluid would help to clarify its applicability in clinical settings.

## Figures and Tables

**Table I.** The base sequences of primers for quantitative real-time PCR.

Primer name	Sequence
Oct4	
Forward	AACGACCATCTGCCGCT
Reverse	CGATACTGGTTCGCTTTCTCT
Sox2	
Forward	GAAAAACGAGGGAAATGGG
Reverse	GCTGTCATTTGCTGTGGGT
Nanog	
Forward	CCTCCTCCCATCCCTCATA
Reverse	TGATTAGGCTCCAACCATACTC
CD44	
Forward	CATCCCAGACGAAGACAGTCC
Reverse	TGATCAGCCATTCTGGAATTTG
GAPDH	
Forward	GGCATCCTGGGCTACACT
Reverse	CCACCACCCTGTTGCTGT
